# I2 Statistic as a Test for Selection Bias in Randomised Controlled Trials

**DOI:** 10.7759/cureus.84769

**Published:** 2025-05-25

**Authors:** Steffen Mickenautsch, Veerasamy Yengopal

**Affiliations:** 1 Faculty of Dentistry, University of the Western Cape, Cape Town, ZAF; 2 Community Dentistry, University of the Witwatersrand, Johannesburg, ZAF

**Keywords:** bias identification, clinical trial appraisal, i2 test, randomized control trial, selection bias, systematic review and meta analysis

## Abstract

This technical report demonstrates that the use of the I^2^ statistic for testing selection bias in single randomised controlled trials (RCTs) has the potential to allow the prevention of false-positive test results, thereby allowing for high test specificity and a high positive predictive value. In addition, the I^2^ statistic provides utility for the in-depth identification of low-level selection bias in RCTs, thus assisting in the avoidance of false-negative test results and possibly for estimating the percentage of trial patients with biased allocation into RCT treatment groups. Future studies to this topic may investigate whether cases with I^2^ estimates above 0%, due to chance rather than selection bias, are possible and, if so, how to distinguish such cases from those with very low bias levels. Future studies may also test the null hypothesis that levels of selection bias are not associated with any over- or underestimation of the true effect estimates of RCTs.

## Introduction

The I^2^ statistic was originally developed to describe the proportion of total variance in the estimates that is due to heterogeneity among trials included in an outcome meta-analysis [1]. The I^2^ point estimate ranges between 0% and 100%, with 0% indicating no heterogeneity beyond the play of chance [2].

In 2014, Clark et al. observed that imbalances in baseline variable measurements between treatment groups in a randomised controlled trial (RCT) are reflected by an increased I^2^ point estimate when pooled with other RCTs in a baseline variable meta-analysis. It was further noted that such an increase might be due to problems with the randomisation process, specifically selection bias [3]. Selection bias deviates from the true effect estimate in RCTs when patients with characteristics conducive to treatment success are not allocated randomly to treatment groups [4].

Based on these observations, Hicks et al. developed a simple test for identifying selection bias in an outcome meta-analysis. The test comprises of the inclusion of baseline measurements from two treatment groups of several RCTs into a fixed-effect baseline variable meta-analysis of continuous data and computation of the t-statistic per RCT, followed by the stepwise removal of RCTs with the largest t-statistic from the baseline variable meta-analysis until I^2 ^= 0%, and subsequent repetition of the outcome meta-analysis without the excluded RCTs [5].

Following the same observations by Clark et al. [3], Mickenautsch and Yengopal modified the test by Hicks et al. [5] for the purpose of identifying selection bias in single RCTs [6-8]. To test a single RCT for selection bias, the mean values with standard deviation (SD) and sample size for baseline variables that are highly predictive for the measured trial outcome are extracted from the trial report for the two treatment groups. The minimum/maximum range of the extracted baseline variable measurements is estimated. In line with the range and the sample size per group, two bias-free simulated comparator trials (SCTs) are generated following the method presented in Appendix 1 and as reported in detail elsewhere [6,8]. The generated values of both SCTs are entered into a fixed-effect meta-analysis for continuous data, with the mean difference (MD) as the outcome measure, and pooled using the inverse variance method. The resulting 0% I^2^ point estimate is noted. The extracted baseline measurements of the RCT are added and the meta-analysis repeated. A resulting 0% I^2^ point estimate indicates the absence and any I^2^ > 0% point estimate the presence of selection bias in the tested RCT.

This technical report has four objectives: to present the mathematical basis for the I^2^ statistic for identifying selection bias in RCTs, to demonstrate why the I^2^ test for single RCTs may not yield false positive results, to show how the test enables the identification of small selection bias presence in RCTs, and to suggest how the extent of selection bias may be estimated.

This manuscript has been published as a preprint in Authorea (www.authorea.com) on May 15, 2025 (https://www.authorea.com/doi/full/10.22541/au.174733690.02299533/v1).

## Technical report

Mathematical basis of the I^2^ statistic

The I^2^ statistic is derived by calculating the study effect estimates (y_i_) and standard errors (SE_i_) - with the former being weighted by the later - from data (s_A/B_ = sample standard deviation of the treatment group A/B; s_i_ = sample standard deviation of the study; n_i_ = study sample size; x_A/B_ = baseline variable mean value of the treatment group A/B) of trials that are included in a meta-analysis:

$(1)y_{i}=\bar{x}_{A}-\bar{x}_{B}$

$(2)s_{i}=\sqrt{s^{2}_{A}}+s^{2}_{B}$

$(3)SE_{i}=\frac{s_{i}}{\sqrt{n_{i}}}$

The study effect estimates (y_i_) and standard errors (SE_i_) form the basis for calculating Cochrane’s Q statistic (with w_i_ = study weight; x_i_ = generic inverse-variance weighted average) [9]:

$(4)\omega_{i}= \frac{1}{SE^{2}_{i}}$

$(5)\bar{x}_{i}=\frac{\sum_{\omega_{i}y_{i}}^{}}{\sum_{\omega_{i}}^{}}$

$(6)Q = \sum_{i=1}^{k}\omega_{i}\left( y_{i} -\bar{x_{i^{}}}\right)^{2}$

From Cochrane’s Q (including: df = degrees of freedom; k = number of studies included in the meta-analysis), Higgins and Thompson (2002) derived the I^2^ statistic [1]:

$(7)df = k - 1$

$(8)I^{2} = \frac{Q-df}{Q}100\%$

**Figure 1:**
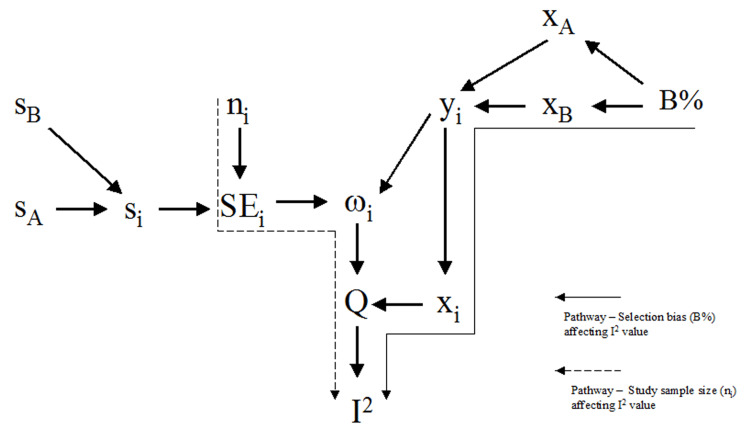
Causal pathways of the selection bias and sample size effect on the I2 (%) value w_i _= study weight; x_i_ = generic inverse-variance weighted average; y_i_ = difference in baseline values between groups /study estimate; SE_i_ = standard error; s_i_ = sample standard deviation; n_i_ = sample size; x_A/B_ = baseline variable mean value of the treatment group A/B; B% - percentage of biased allocated subjects into treatment groups

From these calculation steps, the level of bias (B%) and the study sample size (n_i_) affect the I^2^-point estimate via two separate causal pathways (Figure 1). While the former is affected by the percentage of biased allocated subjects into treatment groups and is thus essential for the selection bias test, the latter is not affected by bias and therefore constitutes a confounding factor.

The level of selection bias (B%) affects the I^2^-point estimate by increasing the difference between the sample means (1) and thus enlarging the study y_i_ estimate. A larger estimate increases the generic inverse-variance weighted average (5), which in turn increases Cochrane’s Q statistic (6) and thus the I^2^-point estimate (8).

Rücker et al. (2008) confirmed in a simulation study that the I^2^-point estimate increases with the number of study subjects [10]. The study weight (w_i_) is defined by the study standard error (SE_i_) according to Equation (4) and the standard error (SE_i_ ) in turn by the study sample size (n_i_) (3). Therefore, the larger the samples size, the smaller the standard error (SE_i_) and the larger the study weight (w_i_). A larger study weight (w_i_) increases Cochrane’s Q statistic and consequently the I^2^ point estimate. Rücker et al. (2008) demonstrated this effect by artificially inflating the sample size in a random-effects meta-analysis, resulting in the I^2^-point estimate tenting to 100% [10].

Prevention of false-positive test results

Due to the confounding effect of the study sample size (n_i_) on the I^2^-point estimate, the I^2^ statistic has been assumed to be of limited use in assessing heterogeneity [10]. Nevertheless, Mickenautsch and Yengopal (2024) observed that the I^2^-based result of the selection bias test is not affected by the sample size when a test threshold of I^2^ = 0% for bias absence and I^2^ > 0% for bias presence is set [2].

A total absence of bias (B = 0%), due to strict random allocation of subjects into treatment groups, prevents in principle an imbalance of baseline variable values beyond chance and results in a zero y_i_ estimate:

$y_{i} = \bar{x}_{A}-\bar{x}_{B} = 0$

A zero y_i_ estimate, in turn, results in a zero weighted average (x_i_) that will cause the Q statistic to be zero; also:

$\bar{x}_{i}=\frac{\sum_{\omega_{i}0}^{}}{\sum_{\omega_{i}}^{}}= 0$

$Q=\sum_{i=1}^{k}\omega_{i}\left( 0-0 \right)^{2} = 0$

A zero Q statistic will cause division by zero during calculation of the I^2^-point estimate and thus will leave its value undefined (conventionally signified by a zero value):

$I^{2}=\frac{0-df}{0}= undefined$

It is notable that division by zero occurs regardless of how large the sample size (n_i_) is and therefore regardless the level of standard error reduction. Such circumstances prevent the selection bias test from generating false-positive test results, due to the confounding effect of n_i_, thus assuring high test specificity and high positive predictive value (PPV).

Identification of low selection bias presence

Any y_i_ estimate larger than zero will be multiplied by a sample size-dependent study weight (w_i_) (5). This will increase Cochrane’s Q (6) and subsequently the I^2^-point estimate (8).

However, when the percentage of biased allocated subjects into treatment groups is low, sample sizes that are realistically used in RCTs may not sufficiently reduce the study standard error to raise the I^2^-point estimate detectably above zero, particularly when common statistical software, such as Review Manger by the Cochrane Collaboration, is used. In such cases, an I^2^-point estimate somewhat slightly above zero may still be reflected as 0% by the software, thus generating a false-negative test result. However, extreme (albeit unrealistic) artificial inflation of the sample size in the baseline variable meta-analysis will increase the point estimate visibly >0%.

For example, in a simulated baseline variable meta-analysis, including a test trial with only 10% biased subject allocation, presented in Appendix 2, the original group sample (n_i_ = 100) size was artificially inflated to n_i_ = 36 000 for all trials, which increased the original 0% point estimate to I^2^ = 50%. The provided example calculations show that the original sample size generated a Q-statistic < df, resulting in an even negative I^2^ point estimate. Because negative values of I^2^ are put equal to zero [11], the forest plot, generated with the Review Manager (version 5.0.24) software, reflected such a point estimate as 0%, accordingly. By contrast, the inflated sample size generated a Q-statistic > df, which was more than sufficient to increase the point estimate > 0%, thus providing a correct true-positive test result.

Estimation of the selection bias extent

**Figure 2:**
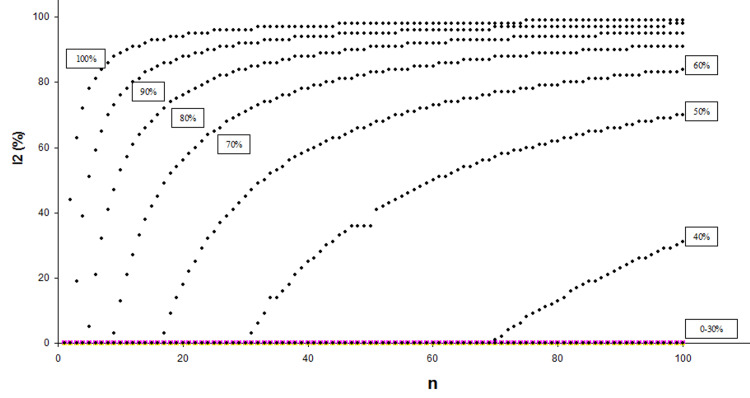
I2-point estimate (%) and sample size (n) relationship at different bias levels (%) 0-100% = percentage of biased allocated subjects into treatment groups

A baseline variable fixed-effect meta-analysis with two SCTs and one test trial at five different variable ranges were simulated using the Review Manager (version 5.0.24) software. The applied simulation method is presented in detail in Appendix 1. In this simulation, the study sample sizes (n_i_) were stepwise increased from 1 to 100, for the percentages of biased allocated subjects in treatments group A and B (B%): 0%, 10%, 20%, 30%, 40%, 50%, 60%, 70%, 80%, 90%, and 100%, each. The I^2^-point estimate was recorded (Appendix 3) and plotted in a scatter plot (Figure 2) per sample size and bias level. In Figure 2, a varying relationship between the I^2^-point estimate and study sample size (n_i_), unique for each bias level (B%), can be observed.

Accordingly, when the I^2^-point estimate was obtained specifically for n_i_ = 10, 50, and 100 at five different minimum/maximum ranges (1.00-5.00, 1.00-10.00, 1.00-15.00, and 1.00-20.00) of the baseline variable for bias levels 40-100%, as well as for bias levels 0-30% with artificially increased sample sizes n_i_ = 5,000, 18,000, and 36,000, certain I^2^-point estimate limits were identified that were specific for each of the 11 different bias levels (Table 1). In this regard, it was taken into account that the minimum/maximum ranges of baseline variables affect the study y_i _estimates and thus the subsequent I^2^ value [7].

**Table 1:** I2 (%) values related to the extent of bias and sample size n_i_ = study sample size; B% = bias levels / percentage of biased allocated subjects into treatment groups

n_i_	B%										
	0	10	20	30	40	50	60	70	80	90	100
10	0	-	-	-	0	0	0	1-40	41-80	41-80	81-90
50	0	-	-	-	0	1-40	41-80	81-90	90-94	>94	>94
100	0	-	-	-	1-40	41-80	81-90	90-94	>94	>94	>94
5,000	0	0	41-85	80-98	-	-	-	-	-	-	-
18,000	0	1-40	80-98	98-99	-	-	-	-	-	-	-
36,000	0	41-85	80-98	98-99	-	-	-	-	-	-	-

These unique I^2^-point estimate limits at the three specified sample sizes for B = 0-30% (n_i_ = 5,000, 18,000, and 36,000) and for B = 40-100% (n_i_ = 10, 50, and 100) (perhaps together with a color coding system as suggested in Appendix 3) may prove useful for estimating the level of bias in a RCT, i.e., for estimating the percentage of subjects that were non-randomly (biased) allocated to the treatment groups. For example, if the pooling of baseline variable values yields an I^2^-point estimate of 0%, 40%, and 72% for sample sizes 10, 50, and 100, respectively, then it may be estimated, according to the limits presented in Table 1, that allocation to treatment groups was biased for between 41% and 50% of all trial subjects.

## Discussion

The I^2^ statistic is not the only method for measuring heterogeneity in meta-analysis; other statistics, such as the t(tau)^2^- statistic, are available [10]. All statistics have been originally developed for use in outcome meta-analyses, and none were specifically as a basis for bias testing in baseline variable meta-analyses. Rücker et al. (2008) have argued that the clinical relevance of heterogeneity should be the basis for deciding whether to pool treatment estimates in a meta-analysis or not and that t(tau)^2^ is the appropriate statistic for this purpose, not I^2^ [10]. Unlike I^2^, the t(tau)^2^-statistic is measured on the same scale as the treatment outcome and describes underlying between-study variability. Furthermore, it does not increase with the number of studies included in a meta-analysis (k) or with the study sample size (n_i_). By contrast, Higgins et al. (2003) have argued that the I^2^ statistic is preferable because it does not depend on the treatment effect scale of one particular trial and thus can be directly compared between different meta-analyses with different types of outcome data, such as odds ratio (OR) and mean difference (MD) [11].

It is our opinion that these arguments are not relevant when I^2^ is used in baseline meta-analyses for selection bias testing. In such circumstances, the calculated I^2^ value is not utilized for assessing between-study heterogeneity but to signify whether selection bias is present (I^2^ > 0%) or not (I^2^ = 0%). Such testing relies on the premise that the I^2^ statistic indicates the proportion of total variance in the estimates that is due to heterogeneity and not measurement error [1] and that no such heterogeneity beyond chance should exist between baseline variable values of two treatment groups when patient allocation into these groups was truly random [3]. Whether the t(tau)^2^-statistic could similarly be used for selection bias testing remains a topic of future research. The current preference of the I^2^ statistic in selection bias testing is based on the fact that Hicks et al. (2018) pioneered this statistic for bias testing purposes [5] and that it is currently the most popular tool for heterogeneity measurement, is included in most computer programmes for meta-analyses, and is therefore most readily available than other types of heterogeneity measures, such as t(tau)^2^ [12].

Based on our applied simulation method (Appendix 1), we generated a total absence of selection bias as an ideal situation under the condition of a zero y_i_ estimate. The possibility of y_i_ > 0 purely due to chance may exist and thus provide the mathematical basis for false-positive test results. Future simulation studies should establish the level of an inflated sample size required for I^2^ > 0% cases when selection bias (B = 0%) is absent but the y_i_ estimate being larger than zero and to distinguish such cases from those with simulated low bias levels at B > 0 to up to 30%.

Unlike in other simulation studies [10], we did not adjust the y_i _estimate when inflating the sample size (n_i_). Bias testing required a fixed effect meta-analysis [5] and not a random-effect model. Furthermore, because an I^2^ > 0% point estimate will signify the presence of selection bias regardless its value, any y_i_ adjustment would only be warranted if it were possible to correlate the y_i_-dependent I^2^-point estimate with any over- or underestimation of the trial outcome estimate.

Our findings suggest that the percentage of trial subjects allocated in a biased way (B%) may be estimated from the I^2^-point estimate values established at several sample sizes (Table 1, Appendix 3). However, it is currently not possible to estimate from the established B% levels how much such bias would divert the reported trial outcome from the true treatment effect. Future meta-epidemiological studies may investigate the relationship between B = 0-100% levels identified in real world RCTs and their reported RCT effect estimates. Any observed statistically significant correlation between the two would provide reason to reject the null hypothesis that B% levels are not associated with any over- or underestimation of true trial outcomes. If the null hypothesis is rejected, further meta-epidemiological studies may establish the actual extent of such over- or under estimation per B% level in RCTs across various fields of medicine. 

## Conclusions

Our technical report demonstrated that the I^2^ statistic as a selection bias test for single RCTs has the potential to prevent false-positive test results, thereby allowing high test specificity and a high positive predictive value for identifying low-level selection bias in RCTs and estimating the percentage of trial patients with biased allocation into RCT treatment groups. Future studies should investigate whether cases with I^2^ estimates above 0% due to chance but without selection bias are possible and, if so, how to distinguish such cases from those with very low bias levels. Future studies should also test the null hypothesis that levels of selection bias are not associated with over- or underestimation of the true RCT outcomes.

## Appendices

All appendices and data are made fully available without restriction and can be freely downloaded via link: https://data.mendeley.com/datasets/cpwp96hwn2/1.
